# The Building Educators’ Skills in Adolescent Mental Health Training Program for Secondary School Educators: Protocol for a Cluster Randomized Controlled Trial

**DOI:** 10.2196/25870

**Published:** 2021-02-24

**Authors:** Belinda L Parker, Cassandra Chakouch, Mirjana Subotic-Kerry, Philip J Batterham, Andrew Mackinnon, Jill M Newby, Alexis E Whitton, Janey McGoldrick, Nicole Cockayne, Bridianne O'Dea

**Affiliations:** 1 Black Dog Institute Randwick Australia; 2 Faculty of Medicine University of New South Wales Kensington Australia; 3 College of Health and Medicine Australian National University Canberra Australia; 4 Faculty of Science University of New South Wales Kensington Australia

**Keywords:** mental health training, schools, teachers, educators, mental health, student mental health, secondary school

## Abstract

**Background:**

In Australia, secondary school educators are well positioned to recognize mental illness among students and provide support. However, many report that they lack the knowledge and confidence to do so, and few mental health training programs available for educators are evidence based. To address this gap, the Black Dog Institute (BDI) developed a web-based training program (Building Educators’ Skills in Adolescent Mental Health [BEAM]) that aims to improve mental health knowledge, confidence, and helping behaviors among secondary school educators in leadership positions. A pilot study of the training program found it to be positively associated with increased confidence and helping behaviors among educators and reduced personal psychological distress. An adequately powered randomized controlled trial (RCT) is needed.

**Objective:**

The primary objective of this cluster RCT is to evaluate the effectiveness of the BEAM program for improving educators’ confidence in managing student mental health. The trial will also evaluate the effect of the BEAM program in increasing educators’ frequency of providing help to students and improving their mental health knowledge and reducing educators’ psychological distress and stigma toward students with mental health issues.

**Methods:**

The target sample size is 234 educators from 47 secondary schools across New South Wales, Australia. Four waves of recruitment and enrollment into the trial are planned. Schools will participate in one wave only and will be randomized to the intervention or waitlist control conditions. Participants from the same school will be assigned to the same condition. Assessments will be conducted at baseline, posttest (10 weeks after baseline), and follow-up (22 weeks after baseline) using the BDI eHealth research platform. Intervention participants will receive access to the BEAM program for 10 weeks upon completion of baseline, and the control condition will receive access for 10 weeks upon completion of the follow-up assessment.

**Results:**

Recruitment for this trial began on July 21, 2020, with the first baseline assessments occurring on August 17, 2020. To date, 295 participants from 71 schools have completed baseline. Due to the unexpected success of recruitment in the first 3 waves, the final fourth wave has been abandoned. Intervention participants are currently receiving the program, with follow-up due for completion in March 2021.

**Conclusions:**

This is one of the first RCTs to examine the effectiveness of a web-based adolescent mental health training program for Australian secondary school educators in leadership positions. If found to be effective, this training program will offer a sustainable and scalable delivery method for upskilling educators in caring for students’ mental health.

**Trial Registration:**

Australian New Zealand Clinical Trials Registry ACTRN12620000876998; https://covid-19.cochrane.org/studies/crs-14669208

**International Registered Report Identifier (IRRID):**

DERR1-10.2196/25870

## Introduction

### Background

Over half of all mental illnesses experienced by adults begin before the age of 18 years [1], yet many young people have difficulty recognizing the early signs and symptoms [2,3]. Unfortunately, many of these young people do not actively seek professional help (eg, from school counselors or psychologists) [4-6]. As secondary schooling is compulsory in Australia, educators are well positioned to identify changes in students’ mental health, offer support, and facilitate access to treatment services [7-9]. However, many report that they lack the confidence and knowledge to recognize and respond to mental health issues among their students [7,10,11]. Educators also report that the increased responsibilities and expectations of caring for students’ mental health increases their levels of work-related stress and psychological distress [12,13]. Thus, there is a clear need for mental health training to improve mental health outcomes for students and to reduce personal stress for educators.

Despite the recommendation by government bodies for greater training in adolescent mental health [14,15], there are very few evidence-based training programs available to educators about student mental health. Recent systematic reviews have identified 6 training programs, only 1 of which was assessed in the Australian context (adult and youth versions of Mental Health First Aid [MHFA]) [16,17]. Of the remaining 5 programs, the Mental Health High School Curriculum Guide teacher training program [18,19], the Go-to Educator Training [20], and The Guide Pre-Service Professional Development Program [21] were all assessed in Canada; the Teachers As Accompagnateurs (TAPS) [22] in Haiti; and the African Guide: Malawi Version [23] in Malawi. All programs provided teachers with information about the common adolescent mental health issues, along with associated signs and symptoms, and directed teachers to additional resources and services. Only the MHFA and TAPS programs provided additional information for teachers on how to identify and support students experiencing a mental health crisis. Of the 6 identified programs, only 1 was assessed using a randomized controlled trial (RCT) methodology, with the remaining 5 employing noncontrolled pre-post designs.

Although all 6 programs were shown to be effective for improving mental health knowledge, none have yet been shown to be effective in increasing helping behaviors, for example, recommending or referring a student to seek professional help, or in reducing the distress experienced by educators [24,25]. In addition, these programs are typically delivered through face-to-face, didactic-style workshops, requiring educators to take leave to attend [24,25]. This may be a barrier to large-scale uptake and increase the financial burden for schools, as they are required to find replacement teachers. With the advancement of technology and effective digitally delivered mental health training programs being developed for workplace settings (eg, refer to the study by Gayed et al [26]), there is an opportunity to develop mental health training for educators that is delivered in new ways.

To fill this gap, the Black Dog Institute (BDI) developed the Building Educators’ Skills in Adolescent Mental Health (BEAM) program. BEAM is a web-based training program on adolescent mental health for secondary school educators in leadership positions, such as year advisors, heads of well-being, and principals*.* To accurately identify educators’ training needs, the BEAM program was initially developed in collaboration with an advisory group of teachers in general teaching and leadership roles. The group consisted of 12 school teachers from various secondary schools located in New South Wales (NSW), Australia. BEAM was originally designed for year advisors, given their role in maintaining student engagement and the well-being of an entire cohort of students. They also often act as case managers, linking students with services and resources when needed, and are usually the first point of contact for parents and teachers. However, there is no formal training for this role, and it is performed in addition to their regular teaching duties. Research on workplace mental health has indicated that training programs for managers can improve their mental health knowledge, thereby improving their confidence and increasing their helping behaviors toward their staff’s mental health needs [26-28]. Furthermore, as nonstigmatizing attitudes have been shown to be associated with intentional or actual contact with individuals with known mental illnesses [28,29], improving stigma may also help increase the assistance provided to students. Therefore, the BEAM program aims to model this workplace mental health research [26-28] while also reducing educators’ stigmatizing attitudes toward students with mental illness. BEAM consists of 5 self-paced and self-directed modules on adolescent mental health (Multimedia Appendix 1), including quizzes, blog style story sharing, and case studies. The program also includes informal, nonmandatory, peer coaching activities that encourage participants to meet with a colleague to discuss the program and apply the content to their own school context. By blending web-based content with face-to-face peer learning, the program aims to foster professional relationships, consolidate learning, and facilitate new problem-solving skills [30]. This flexible model allows users to complete the program at a time that is convenient for both the educator and their school without the educator having to take leave to attend face-to-face workshops.

A recent pilot study examined the acceptability of the BEAM program among secondary school year advisors (N=71) from NSW, Australia. After using the program for 6 weeks, the year advisors reported significantly higher levels of self-reported confidence in their ability to care for students’ mental health and lower levels of personal psychological distress. Year advisors also reported an increased frequency of helping behaviors at the 19-week follow-up. However, many of the year advisors did not complete the entire program (59/70, 84%) reporting that the 6-week duration was insufficient, and barriers such as forgetfulness hampered their completion. In preparation for this trial, several modifications were made to the program to increase engagement and completion: program access was extended to 10 weeks, SMS reminders in addition to email reminders were embedded, sequential module completion was removed so that participants can complete the modules in any order, a *module suggestion* function was embedded that encourages the participant to complete modules based on their interest, and the program was optimized for completion on both mobile and desktop devices to increase accessibility. The eligibility criteria for BEAM have also been extended to encompass other leadership roles within the school, including principals, heads of well-being, and directors of pastoral care, among others. This decision was made because these staff members also have responsibilities regarding student well-being in addition to their regular teaching duties and are well placed to influence schools’ policies and possibly enact change. The program is now ready to be evaluated for its effectiveness using an RCT.

### Objectives

The primary objective of this trial is to evaluate the effectiveness of the BEAM program in improving secondary school educators’ confidence in recognizing and responding to their students’ mental health needs. The secondary objective is to assess BEAM’s effectiveness in increasing the frequency of help provided to students, improving educators’ mental health knowledge, reducing educators’ stigma toward others with mental illnesses, and reducing their own psychological distress.

### Hypotheses

The primary hypothesis is that educators who are allocated to receive the BEAM program will report significantly higher levels of confidence at posttest (primary endpoint) when compared with the waitlist control condition and that these effects will be sustained at follow-up. It is also hypothesized that educators who receive the BEAM program will report greater improvements in mental health knowledge, stigma, and psychological distress at posttest and a greater frequency of helping behaviors at follow-up, when compared with those in the control condition.

## Methods

### Design

This study is a cluster randomized controlled effectiveness trial with 2 parallel conditions (the BEAM program and waitlist control), with measurements taken at baseline, posttest (10 weeks from baseline completion), and follow-up (22 weeks from baseline completion). This study protocol was approved by the primary ethics body of the University of New South Wales (UNSW) Human Research Ethics Committee (HREC; HC200257). Approval was also sought and obtained from the NSW Department of Education State Education Research Applications Process (SERAP2020222) to conduct research within government schools and from the Catholic Schools Office Dioceses of Maitland-Newcastle, Canberra-Goulburn (schools located in the Goulburn area only), and Wollongong to conduct research with schools located within their dioceses.

This research project is also guided by a trial management committee consisting of experts in research and trial design and service and program implementation to oversee and provide guidance on the study procedures. This committee meets bimonthly or more frequently on an as-needed basis.

### Participants

#### Inclusion Criteria

To be eligible to participate, secondary school educators must be (1) employed in a school leadership position that includes responsibility for student well-being (eg, year advisors, directors or heads of student well-being, principals, directors of pastoral care, student coordinators, and heads of year); (2) currently working in this role at a government, Catholic, or independent secondary school in NSW, Australia, for the duration of the study; and (3) obtain their principal’s consent for their involvement.

#### Exclusion Criteria

Secondary school educators who participated in the pilot study are not eligible to participate in this trial.

### Sample Size

The target sample size for this trial is 234 participants from at least 47 schools. This calculation is conservatively based on the participation of an average of 5 educators per school and an intraclass correlation of 0.07, which yields a design effect of 1.28. To detect a standardized effect size of 0.50, (a minimum of a moderate effect size is required to warrant future value and benefit of the program) with 80% power and α=.05 (2-tailed), an individually randomized trial would require 64 participants per arm. This number is inflated by the design effect to 82 to allow for clustering, with the recruitment increased to conservatively allow for attrition of up to 30%. This yields a minimum of 117 participants per arm.

### Randomization and Blinding

Cluster randomization at the school level is used to avoid potential contamination and bias effects from other participants, reduce administrative tasks for schools, and enable implementation of the peer-to-peer component of the program. As such, all participants from the same school will be allocated to the same condition and complete the trial at the same time using randomly permuted block randomization with block sizes of 2 and 4. Schools will be assigned to either the intervention or control arm using a 1:1 allocation, stratified by school size (<400 or >400 students) and index of community socioeducational advantage (ICSEA) level (<1000 or >1000) as per a computer-generated randomization schedule. Randomization is conducted by a statistician not involved in the day-to-day running of the trial to avoid influence or bias. The research team will be aware of the allocation once registration is scheduled to begin because they are responsible for providing the link to participants for the intervention or control program study website. Participants will be unaware of which group they are allocated to during registration and baseline. Upon completion of baseline, they will be informed, via email, of their allocation. This is because the intervention participants will receive immediate instructions and access to the program, and the waitlist control participants will be asked to wait for their next survey.

### Recruitment

Multimedia Appendix 2 outlines the recruitment, randomization, and procedure for this trial.

A passive approach to recruitment is being undertaken using study advertisements. The advertisements are being placed on the BDI’s website and social media channels (Facebook, LinkedIn, and Twitter), within BDI newsletters, and circulated to BDI mailing lists and contacts. The study is also being advertised in the NSW Health School-Link e-newsletters (an NSW Health service that connects schools with local mental health services), Teacher Magazine, and Catholic Diocese bulletins. After viewing the study advertisements, interested educators are directed to a web-based expression of interest form on the BDI website, which collects their name, school, suburb, email, and role at their school. Once registered, prospective participants are encouraged to share the study information with their colleagues to increase cluster sizes and encourage peer-to-peer interaction. To promote a representative sample from metropolitan, regional, and rural locations, participation will be open to all government, Catholic, and independent schools within the Australian state of NSW for which we have ethical approval.

### Consent

#### Principal Consent

After expressing interest, the educators are emailed the study information. Interested participants consult their principal to obtain a signed letter of support. The school and its educators are randomized after the letter of support is received by the research team. Only 1 signed letter of support is required per school.

#### Educator Consent

Educator consent is obtained online. Prospective participants provide consent by confirming the declaration statements in the web-based participant information sheet and consent form (PISCF). Participants can download the PISCF before providing consent, and they are emailed a copy for their records.

#### Withdrawal of Consent

Participants can withdraw consent at any time without providing a reason by contacting the research team, replying to any email communication with the word *withdraw*, or completing the withdrawal form located within the PISCF. When a participant withdraws, all study data are retained, but no further data are collected, and all study communication ceases.

### Procedure

#### Registration

This trial will include 4 waves of registration and enrollment. A predetermined cut-off date indicates which wave a school participates in, as determined by the date on which the letter of support is received. Using waves ensures that data collection does not occur during summer school holidays, allows flexibility for when schools enroll in the trial based on their schedule, and ensures that participants from the same school commence the trial concurrently. Once all registered participants from a single school commence the baseline survey, no other prospective colleagues from that school can register. This prevents the influence that knowledge of group allocation might have on future participant enrollment. To avoid disappointment, educators are asked to tell their colleagues about the study at the time of recruitment.

Participants are sent an email directing them to the study registration website 1 week before the scheduled baseline start date. Here, they are asked to confirm their eligibility, register their personal details (including their name, school, and email), provide consent, and create a study account. They have the option to enter their mobile phone number to receive SMS notifications and reminders. Once completed, they await further instructions and access to the baseline survey.

#### Baseline, Posttest, and Follow-Up Assessments

On the day each wave is due to begin, participants receive an email (and optional SMS) inviting them to complete the baseline survey. The survey is accessible for 7 days, and participants who do not complete it are automatically withdrawn. This process is repeated for the 10-week and 22-week assessments. All participants receive 2 email reminders (and 2 optional SMS reminders) for the survey completions.

### Intervention Condition

The BEAM program is a web-based training program accessible on any internet-enabled device. Each of the 5 modules consists of information, web-based interactive activities, and downloadable resources related to adolescent mental health. In this trial, participants can complete the 5 program modules in any order; however, an initial module is suggested to participants based on their response to the *module recommendation* question in the baseline survey. This question presents participants with a list of 10 topics (such as *signs and symptoms* and *about my role*) that are linked to the learning objectives of each module. Participants are then asked to rate the 3 they are most interested in learning about from 1 (*most interested*) to 3 (*least interested*). The program then recommends that they begin with the module corresponding to their first choice. Each module includes an optional peer coaching activity that asks participants to meet with a colleague from their school to discuss focus questions, practice, and apply program learnings. Participants then submit their responses to the focus questions through the program. The research team then send standardized feedback via email within 3 business days. Participants who do not have another colleague from their school taking part in the trial can either complete the peer coaching themselves, talk to another colleague who is not taking part, or skip the activity, as it is not mandatory. No other program activities are reviewed by the research team. All participants receive access to the program at no cost for 10 weeks, and it is estimated that the full program takes approximately 6.5 hours to complete. Participants can complete the program at their own pace; however, they are recommended to undertake 1 module per fortnight. Given the current COVID-19–related school closures and physical distancing guidelines, participants are encouraged to complete the peer coaching activities via teleconference or phone. Participants will receive fortnightly email reminders to use BEAM and optional SMS reminders. All program use data are collected by the BDI web-based eHealth research platform hosted on the UNSW servers.

### Control Condition

This study uses a waitlist control condition. Participants in the control condition will receive access to the intervention at no cost immediately after they complete the follow-up survey (22 weeks post baseline). If they do not complete the follow-up survey, they will receive access immediately after the survey has closed. They will receive access to the full program whether they have not completed the survey or not.

### Reimbursements

Participants in both conditions will receive an Aus $15 (US $11.59) e-gift voucher to thank them for their time and completion of the posttest survey. They will also receive an Aus $15 (US $11.59) e-gift voucher after completing the follow-up survey. All e-gift cards will be emailed within 5 working days of a participant completing the survey and will be issued through GiftPay.

### Outcome Measures

Table 1 shows the administration schedule of measures.

**Table 1:** Schedule of outcome measures.

Measure	Data collection timepoint		
	Baseline	Posttest (10 weeks)	Follow-up (22 weeks)
Demographics and background	✓^a^	—^b^	—
Experience of mental health	✓	—	—
Self-care	✓	✓	✓
Experience in mental health training	✓	—	—
School factors	✓	✓	✓
Impact of COVID-19 on helping behaviors	✓	✓	✓
Perceived mental health knowledge and awareness	✓	✓	✓
Mental health knowledge	✓	✓	✓
Stigma	✓	✓	✓
Confidence	✓	✓	✓
Helping behaviors	✓	✓	✓
Psychological distress	✓	✓	✓
Module recommendation question	✓	—	—
Barriers to use	—	✓	—
Program satisfaction	—	✓	—
Process evaluation	—	✓	—
Program impact on future behaviors	—	—	✓

^a^Indicates the timepoint measure is administered.

^b^Indicates the measure is not administered at that timepoint.

#### Primary Outcome Measure

The primary outcome for this trial is educators’ confidence in recognizing and responding to students’ mental health needs. This is measured using an adapted version of the confidence to recognize, refer, and support subscale from the study by Sebbens et al [31]. Participants are asked to rate how confident they feel about a set of 15 scenarios (eg, *recognizing a student with mental health problems*) using a 5-point Likert scale ranging from 1 (*not at all confident*) to 5 (*very confident*). Mean total scores are calculated to represent participants’ self-reported confidence in managing their students’ mental health needs. Total scores can range from 15 to 75, with higher scores indicating greater levels of confidence. Scores will be compared over time and between the intervention and control arms at each time point.

#### Secondary Outcome Measures

##### Helping Behaviors for Mental Health

A modified version of the Help Provided to Students Questionnaire by Jorm et al [16] is used to assess the frequency of helping behaviors for mental health among educators. Participants indicate how often they have engaged in 13 helping behaviors (eg, *spent time calming a student down*) during the past 2 months. This is answered using a 4-point scale (*never*, *once*, *occasionally*, *frequently*). Items are then summed to create a total score (range: 15 to 60), with higher scores indicating a greater frequency of helping behaviors.

##### Perceived Mental Health Knowledge and Awareness

This is assessed using the Perceived Knowledge and the Perceived Awareness subscales from the Mental Health Literacy and Capacity Survey for Educators [32]. Participants are asked to rate their level of perceived knowledge on a set of 4 statements (eg, *how would you rate your knowledge of the signs and symptoms of student mental health issues*) from 0 (*not at all*) to 4 (*extremely*). Items are then summed to create a total score (range: 0-16), with higher scores indicating greater knowledge of mental health.

For the awareness subscale, participants are asked to rate their level of perceived awareness on a set of 5 statements (eg, *how would you rate your awareness of the risk factors and causes of student mental health issues*) from 0 (*not at all*) to 4 (*extremely*). Items are then summed to create a total score (range: 0-20), with higher scores indicating greater awareness of mental health issues.

##### Mental Health Knowledge

This consists of 2 constructs: mental health literacy and the recognition of common mental illnesses. These 2 constructs are measured using an adapted version of the 12-item Mental Health Knowledge Schedule (MAKS; [33]) and 2 vignettes adapted from the study by Jorm and Wright [34]. To measure mental health literacy, participants are asked to rate how much they agree with the first 6 items on the MAKS (eg, *Most students with mental health problems want to complete their schooling*) using a Likert scale ranging from 1 (*strongly disagree*) to 5 (*strongly agree*). Items are then summed to create a total score (range: 6-30), with higher scores indicating higher mental health literacy. Recognition of common mental illnesses is assessed using the remaining 6 items from the adapted MAKS (items 7-12), where participants are asked to rate whether they believe the conditions of depression, stress, grief, anxiety, self-harm, and substance misuse are mental illnesses using a Likert scale ranging from 1 (*strongly disagree*) to 5 (*strongly agree*). Participants are also asked to read 2 vignettes adapted from the study by Jorm and Wright [34]. These vignettes describe 2 adolescents with depression or anxiety, and the participants are asked to indicate which mental illness they believe the scenario depicts (free response).

##### Stigma

A modified version of the Personal Stigma subscale from the Depression Stigma Scale from Griffiths et al [35] is used to measure stigma toward mental health illnesses. Participants are asked to rate how much they agree with 9 statements (eg, *students with a mental illness could snap out of it if they wanted*) using a Likert scale ranging from 1 (*strongly disagree*) to 5 (*strongly agree*). Items are then summed to create a total score (range: 9-45), with higher scores indicating greater levels of stigma.

##### Psychological Distress

The Distress Questionnaire-5 (DQ-5) [36] is used to assess the personal psychological distress of educators. Participants are asked to rate how frequently, in the past 6 weeks, they have experienced 5 symptoms (eg, *Thinking back over the past 6 weeks, how often have you felt hopeless*). Answers are given using a 5-point scale ranging from 1 (*never*) to 5 (*always*). Items are then summed to create a total score (range: 5-25), with higher scores indicating greater levels of psychological distress. The DQ-5 has high internal consistency and convergent validity [36,37].

#### Supplementary Measures

##### Demographics and Background Factors

Participants are asked to provide their name, age, gender identity, whether they identify as Aboriginal or Torres Strait Islander, current role at their school, duration of employment at their current school (years), overall experience as an educator (years), and experience in their current role (years) at baseline.

##### Experience of Mental Illness

Participants are asked to indicate whether they have had a personal, family, or close friend experience a mental illness (answered *yes* or *no* or *prefer not to answer*).

Participants are also asked to rate how frequently they engage in self-care. This is to be answered on a 6-point scale (*never*, *less than once a month*, *once a month*, *a few times a month*, *weekly*, or *daily*).

##### Experience in Mental Health Training

Participants are asked to rate how important they believe mental health training is for educators (answered 0, *not at all important* to 4, *extremely important*), how they rate their level of mental health training (answered 0 *none to date* to 3 *extensive training*), the mental health training programs they have completed (free response), and how confident they are that an online program can meet their training needs (answered 0 *not at all confident* to 4 *extremely confident*).

##### School Factors

Participants are asked to indicate their school location (*metropolitan*, *regional*, or *rural*), school type (*government*, *Catholic*, or *independent*), and whether their school is same sex (answered *yes* or *no*). Participants are also asked to indicate whether their school has a student well-being policy (answered *yes* or *no*) and staffing roles to support students’ mental health (answered *yes* or *no*). Participants are also asked to rate the degree to which mental health is their school’s priority (answered 0 *not a priority* to 4 *high priority*) and how responsible they feel for their students’ mental health and well-being (answered 0 *not at all responsible* to 4 *completely responsible*). Participants are also asked to indicate, on average, the hours per week they spend supporting students’ mental health needs and how supported they feel by their colleagues, supervisor or employer, workplace, friends, and family (answered 0 *not at all* to 4 *extremely*).

##### Impact of COVID-19 on Helping Behaviors

In response to COVID-19, participants in this trial are asked questions regarding helping behaviors that may have changed due to the pandemic. The first 3 questions, “Have you reached out to students in a way that is different than you have done so in the past because of COVID-19? (eg, connecting via technology)?”, “Have you implemented a service, program, or educational information session about mental health?”, and “Are there any other ways you have responded to your students’ mental health that isn’t covered here?” will require a *yes* or *no* response. If a participant answered *yes* to the latter 2 questions, they were asked to specify (free response). Participants are also asked how often then have contacted their students about mental health using technology (email or school e-learning platform); this is answered using a 4-point ordinal scale from 1 (*never*) to 4 (*frequently*).

#### Measures for Intervention Participants Only

The following measures will be obtained only from participants assigned to BEAM and will be used in subsequent research into factors that may moderate or mediate outcomes.

##### Program Use

Program use will be measured by the number of completed lessons (maximum of 27), collected automatically by the eHealth research platform.

##### Barriers to Use

Program barriers will be identified using a 13-item list at posttest.

Participants will be asked to report if they experienced any of the listed barriers throughout the trial (eg, *Forgot about it* and *Didn’t have enough time*); this is answered as *yes* or *no*. If a participant answers *yes* to the 13th item *Other not listed above (Please specify)*, a mandatory free response textbox will appear for the participant to provide more detail.

##### Program Satisfaction

Participants are asked to rate the extent to which they agreed with a set of 14 statements about the BEAM program (such as *I enjoyed using BEAM* and *the content was easy to understand*). This is answered using a 5-point Likert scale ranging from 1 (*strongly disagree*) to 5 (*strongly agree*).

##### Process Evaluation

Participants are asked whether they completed the peer coaching activities with a colleague (answered *yes* or *no*), and if yes, how frequently they met (*more than once a week*, *about once a week*, *about once a fortnight*, and *about once a month*) and whether they found these activities to be valuable (answered *yes*, *no*, or *not appropriate*). Participants are also asked “What could we do to improve the program,” “Is there any content or topics you would have liked to have been covered in the training?”, and “Is there anything else you would like to say about the program and its value for you?” (all answered with free responses). Finally, participants are asked what device they completed the program on (eg, laptop, tablet, or mobile).

##### Program Impact on Future Behaviors

Participants are asked to indicate whether the program content or resources were shared with other school staff and if any well-being programs had been implemented during the trial period (answered *yes* or *no*). If they answer *yes,* participants are asked to provide more detail (free response).

#### Statistical Methods

The primary analysis will use a mixed model repeated measures analysis of variance (MMRM), accounting for repeated assessments within individuals and a random effect to account for clustering within schools. Models will include the factors time, condition (intervention vs control), and their interaction, with the critical test of effectiveness being planned contrasts of this interaction from baseline to postintervention (the trial primary endpoint) and follow-up (secondary endpoint). An unconstrained variance-covariance matrix will be used to accommodate within-participant effects. The method of Kenward and Roger [38] will be used to estimate the degrees of freedom for tests of all effects. Any baseline variables identified as substantially imbalanced between groups will be added to the models on an exploratory basis to confirm the robustness of the findings to this imbalance. Where distributional assumptions cannot be satisfied, bootstrapping methods or generalized mixed models (eg, binary MMRM) may be used to confirm the robustness of the findings. MMRM constitutes an intention-to-treat analysis, as it includes all available data under the missing-at-random assumption. Between-group effect sizes will be estimated using the estimated model means and variances.

Secondary and additional outcome analyses will involve contrasts comparing changes from baseline to follow-up analyses of secondary outcomes (mental health knowledge, stigma, helping behaviors, and psychological distress) from baseline to other occasions of measurement, using an MMRM approach, as described above. If the intervention is found to be effective, exploratory analyses will examine evidence for moderation effects, that is, whether the intervention was more effective for certain subgroups of the sample. This may include teacher attributes such as gender or age and school characteristics such as ICSEA status.

## Results

Approval was obtained from the primary ethics body (UNSW HREC) on April 21, 2020, SERAP on July 21, 2020, Maitland-Newcastle Catholic Diocese on May 27, 2020, Canberra-Goulburn Catholic Diocese on June 12, 2020, and Wollongong Catholic Diocese on August 4, 2020. Recruitment of educators started on July 21, 2020, and the baseline for waves 1, 2, and 3 are complete. To date, 465 educators have expressed interest in participating in the trial. In total, 308 educators have registered and 295 have completed baseline, representing 71 schools. Due to the unexpected success of recruitment in the first 3 waves, the decision has been made to not go ahead with the final fourth wave. Intervention participants are currently receiving the program with follow-up due for completion in March 2021. It is planned that the results will be presented at both national and international conferences and submitted to peer-reviewed journals. The results will also be disseminated to stakeholders through reports and presentations and on the BDI website.

## Discussion

### Principal Findings

This protocol describes the RCT of the BEAM program, a study that aims to evaluate the effectiveness of a new web-based mental health training program for secondary school educators in leadership positions. Through the provision of mental health information and interactive activities, the BEAM program aims to improve educators’ knowledge of adolescent mental health. It is anticipated that their confidence in managing their students’ needs and the frequency of help provided will thereby increase, whereas their stigma toward mental ill-health and their own levels of psychological distress will reduce.

There remains a significant lack of available evidence-based mental health training programs for educators [24,25] and few high-quality studies that have evaluated adolescent mental health education for educators. Of those identified in the study by Anderson et al [24], only 2 studies were conducted as RCTs, whereas the others used a pre-post design with no comparator. Furthermore, only 1 study identified in the study by Anderson et al [24] was conducted in Australia [16], which significantly limits the quality of training options for educators containing information relevant to their education system. It is recommended that educators are provided with professional development opportunities regarding adolescent mental health [14,15]; however, there are few good-quality and relevant programs available. The BEAM program may help fill this training gap if shown to be effective, providing more education options for Australian secondary school educators that have been formally evaluated. To our knowledge, this is the first RCT assessing a web-based training program for educators, which also measures changes in helping behaviors and psychological distress. This is important, given the ongoing impact of COVID-19 on education delivery and school closures, which has likely added to both student and teacher stress. Physical distancing guidelines have caused the cessation of most face-to-face training, meaning the options for educators are now further limited and other methods, such as web-based delivery, are ever more important if they are effective.

If the effectiveness of the BEAM program is demonstrated through this trial, there are significant implications for how mental health training can be delivered to educators. For example, traditional educator training is typically delivered through face-to-face didactic-style workshops that require participants to take leave and have their classroom duties covered by another staff member [24]. By delivering the training online, educators can access the standardized material in a flexible and personalized manner anywhere there is an internet connection. Web-based delivery potentially lowers organization and administration costs when compared with attending face-to-face training, including the financial cost of replacing staff to cover classroom duties or paying for extensive travel for training. Finally, by delivering the training online, educators can easily revise the relevant material at any time by simply logging back into the program. The drawbacks to web-based program delivery are, however, acknowledged, in particular, the full completion of the training and engagement with the content and possible ambiguity for workplaces to determine when it is reasonable for their staff to use work time to complete training. Therefore, it is worthwhile to explore methods to ensure completion and engagement with the content, such as professional accreditation with the NSW Education Standards Authority.

### Limitations and Strengths

There are limitations to this trial that are acknowledged, including the reliance on self-report questionnaires, which may be susceptible to biases. Self-report outcomes, such as confidence, may not match actual educator behavior; however, we will use validated measures where possible and assess teacher reports of actual helping behavior.

Another limitation is that the trial is being conducted among educators who self-selected to participate from within NSW only. The results may not be generalizable to interstate educators or educators who chose not to participate. Although Australia has a national education curriculum, schools are governed by each of the States’ and Territories’ Department of Education, and Catholic schools are governed by the Diocese to which they belong. Each has their own set of frameworks, policies, and rules that guide staffing. For example, there are structural differences between the states of South Australia (SA) and NSW, such that secondary school begins in year 8 in SA and year 7 in NSW. It may also be the case that participating educators are employed at better-resourced schools and have the time to take part. Furthermore, participation is limited to educators from schools for which we have approval from their governing ethical body and could obtain support from their school principal. Not all Catholic Dioceses in NSW granted approval to conduct this research, and gaining principal support might not have been possible for all interested educators.

A further limitation is the use of waitlist control and trial length. The total amount of time a control participant is required to wait without any access to the intervention is 22 weeks, which may affect attrition. We have conservatively estimated a 30% attrition in our target sample size, and a monetary reimbursement will be used to motivate completion of the posttest and follow-up surveys. The waitlist control also only enables the assessment of whether the intervention is more effective than the passage of time. Future follow-up studies to compare the intervention with active controls are needed once effectiveness relative to waitlist control is established.

Despite these limitations, this trial has several strengths, including the RCT design to examine the effectiveness of BEAM and the clustering and randomization at the school level to reduce the risk of bias and contamination. Once a school begins in the trial, no other educators from that school can enroll, ensuring that participants are not influenced by the participation of other staff members from their school. The standardized delivery of the intervention helps maximize the fidelity of the training being delivered to educators, ensuring that all participants receive the same standardized intervention. Other strengths include the suite of measures included to examine a range of outcomes; the long-term follow-up period; targeted recruiting approach to include educators from metropolitan, regional, and rural areas; exploration of possible moderators; and stratification of school variables to account for factors hypothesized to influence the results. If shown to be effective, the assessment of BEAM through an RCT will provide a novel method for delivering mental health training to secondary school educators.

## Appendix

Multimedia Appendix 1 Overview of the Building Educators’ Skills in Adolescent Mental Health Training program.

Multimedia Appendix 2 CONSORT (Consolidated Standards of Reporting Trials) flow diagram that will be used to outline participation through the Building Educators’ Skills in Adolescent Mental Health Training program for secondary school educators. NSW: New South Wales.

## Abbreviations

BDI: Black Dog Institute
BEAM: Building Educators’ Skills in Adolescent Mental Health
DQ-5: Distress Questionnaire-5
HREC: Human Research Ethics Committee
ICSEA: index of community socioeducational advantage
MAKS: Mental Health Knowledge Schedule
MHFA: Mental Health First Aid
MMRM: mixed model repeated measures analysis of variance
NSW: New South Wales
PISCF: participant information sheet and consent form
RCT: randomized controlled trial
SA: South Australia
SERAP: State Education Research Applications Process
TAPS: Teachers As Accompagnateurs
UNSW: University of New South Wales
