# Supplementary material for: The Building Educators’ Skills in Adolescent Mental Health Training Program for Secondary School Educators: Protocol for a Cluster Randomized Controlled Trial
Source: JMIR Res Protoc. 2021 Feb 24;10(2):e25870. doi: 10.2196/25870 (PMC7946581; doi:10.2196/25870)
Supplement: Multimedia Appendix 1 [file resprot_v10i2e25870_app1.pdf]

1. Your role

Lessons
- School and mental health
- Supporting your students
- Identifying who can help

2. Identifying students in need

Lessons
- Common mental health issues
- Causes and risks
- Signs and symptoms
- Recognizing those at risk

3. Taking action

Lessons
- Having a chat
- Facilitating help-seeking
- Modifying education
- Returning to school

4. Managing yourself and others

Lessons
- Respectful communication
- Responding to student needs
- Managing difficult situations

5. Maintaining your wellbeing

Lessons
- Student wellbeing
- Student Resilience
- Looking after yourself
