# Supplementary material for: The Building Educators’ Skills in Adolescent Mental Health Training Program for Secondary School Educators: Protocol for a Cluster Randomized Controlled Trial
Source: JMIR Res Protoc. 2021 Feb 24;10(2):e25870. doi: 10.2196/25870 (PMC7946581; doi:10.2196/25870)
Supplement: Multimedia Appendix 2 [file resprot_v10i2e25870_app2.docx]

Enrollment

Educators express their interest,

N=

Educators excluded:

School counsellor, n=

Outside NSW, n=

Nonapproved Catholic Diocese, n=

No principal Support, n=

School randomization

Wave 2

Schools, n=

Educators, n=

Wave 4

Schools, n=

Educators, n=

Wave 3

Schools, n=

Educators, n=

Wave 1

Schools, n=

Educators, n=

**Allocated to Intervention Arm**

Total schools, n=

Total eligible educators, n=

**Allocated to Control Arm**

Total schools, n=

Total eligible educators, n=

Educators consented, n= representing N schools

Educators consented, n= representing N schools

Allocation

Baseline surveys completed, n=

Baseline surveys not completed, n=

Baseline surveys completed, n=

Baseline surveys not completed, n=

Baseline, N=

Active withdrawals, n=

Active withdrawals, n=

Posttest surveys completed, n=

Posttest surveys not completed, n=

Posttest surveys completed, n=

Posttest surveys not completed, n=

10-week posttest, N=

Follow-up surveys completed, n=

Follow-up surveys not completed, n=

Follow-up surveys completed, n=

Follow-up surveys not completed, n=

22-week follow-up, N=

Analysis
